# Effectiveness of Digital interventions on Mental Health and Psychological Well-being of College and University students

**DOI:** 10.12669/pjms.41.9.12009

**Published:** 2025-09

**Authors:** Farah Rashid, Nusrat Zareen, Tabassum Alvi, Hadiya Rashed Siddiqui

**Affiliations:** 1 Farah Rashid Department of Community Medicine, NUST School of Health Sciences, National University of Sciences and Technology (NUST), H-12 Sector, Islamabad 44000, Pakistan; 2 Nusrat Zareen Department of Anatomy, Watim Medical & Dental College, Rawalpindi, Pakistan; 3 Tabassum Alvi Department of Psychiatry, Avicenna Medical College, Lahore, Pakistan; 4 Hadiya Rashed Siddiqui 3^rd^ Year BDS Student, Islamabad Medical & Dental College, Islamabad, Pakistan

**Keywords:** Digital intervention, Higher education, Mental health, Psychological well-being, Web-based intervention, University students

## Abstract

**Objective:**

The main objective of this study was to determine the overall effectiveness of various digital therapies in treating symptoms of depression, anxiety and general psychological well-being.

**Methodology:**

A systematic literature search of various Databases including PubMed, PsycINFO, Google Scholar, Web of Science & Cochrane library was carried out between the years 2006 to 2024. The data collected was reported following the PRISMA guidelines. The study examines the potential of digital platforms to address mental health issues. Compiling data from 20 carefully selected studies involving 30639 participants, the research compares outcomes for depression, anxiety and overall psychological well-being.

**Results:**

The results reveal significant effect sizes (Hedges’ g: 0.80-0.88), demonstrating the positive impact of digital interventions. These tools not only provide therapeutic and preventive benefits but also improve accessibility. Notably, the overall psychological well-being effect size of 0.88 emphasizes their role in promoting mental health. While the findings support a shift toward digital solutions in student support systems, they also highlight the need for continued investment in refining and integrating these platforms. This analysis advocates for rethinking mental health support in higher education, bridging gaps with innovative digital approaches.

**Conclusion:**

Digital interventions effectively improve students’ mental health by reducing stress, anxiety and depression while enhancing well-being. Their success depends on engagement, design and individual differences. Further research is needed to optimize long-term effectiveness.

## INTRODUCTION

The transition to college or university often marks a pivotal moment in a young person’s life. Alongside newfound independence and academic challenges, students struggle with multifaceted stressors, from social adjustments to financial strains.^1^,^2^ Mental health, an integral component of overall wellbeing, has been a growing concern within the realm of higher education.^3^ The college and university years are vulnerable regarding all the challenges met during the academic years.^4^ Based on a survey published through the American College and University Health Organization, a significant number of students expressed experiencing high levels of anxiety, while a substantial subset reported experiencing such severe depression that it impeded their ability to carry out daily activities. Such statistics are not isolated to any one region but echo globally, underscoring the pressing need to address mental health within the academic milieu.^5^

As a consequence of mental health issues, academic performance can wane, dropout rates may rise and in severe cases, students might face debilitating conditions or even contemplate self-harm. In addition to the individual consequences, the implications may extend to the wider society, negatively effecting the community at large.^6^

In the present era there has been a notable increase in the availability and use of digital mental health resources. These resources include a wide range of technologies, including online treatment platforms and mindfulness applications.^7^ Digital treatments provide several benefits, including enhanced accessibility compared to conventional in-person therapy, increased affordability and the provision of a reassuring amount of anonymity for some individuals. Furthermore, these platforms are designed to accommodate the technologically proficient tendencies of the contemporary cohort of students, hence increasing the likelihood of their active participation and deriving advantages from these platforms.^8^ Nevertheless, similar to any emerging discipline, digital psychological tools exhibit a range in quality and effectiveness. While several interventions are supported by extensive research and clinical competence, others may exhibit limited efficacy or even pose possible risks. The aforementioned variability highlights the need of doing thorough assessments and verification of these instruments, particularly when focusing on a susceptible demographic such as students at universities and colleges.^7^

By utilizing digital interventions without a clear understanding of which tools or methodologies are genuinely impactful, we risk inundating students with ineffective or even detrimental resources. Researchers endorse that institutions, clinicians and policymakers need a consolidated knowledge base to make informed decisions about implementing and recommending these digital interventions.^8^ An evidence-based approach, such as the review proposed, serves as an invaluable tool in this endeavor. By synthesizing data from multiple studies, we can derive more robust conclusions about the overall effectiveness of digital interventions, paving the way for more targeted and effective mental health strategies in higher education settings.^9^ As digital interventions offer a promising avenue for support, it is our academic and ethical responsibility to ensure that these tools are not only available but are also effective and evidence based.

The present review aimed to conduct a complete evaluation and synthesis of existing evidence pertaining to digital mental health treatments specifically designed for students at colleges and universities. The main objective of this study was to determine the overall effectiveness of various therapies in treating symptoms of depression, anxiety and general psychological well-being. Through an extensive review, we seek to measure the usability, acceptability and effectiveness of these digital platforms, categorize the varied types of interventions available, evaluate the post-intervention mental health outcomes and assess the quality of the evidence using the GRADE approach. By doing so, we hope to provide stakeholders with a clear understanding of the digital mental health landscape within higher education settings.

## METHODOLOGY

A systematic literature search of various Databases was carried out between the years 2006 to 2024. The data collected was reported following the PRISMA guidelines. The study examines the potential of digital platforms to address mental health issues. Compiling data from 20 carefully selected studies involving 30639 participants, the research compares outcomes for depression, anxiety and overall psychological well-being.

### Databases Used:

For an in-depth and wide-ranging literature search, we meticulously scoured several databases known for their extensive academic content and relevance to the domains of psychology, medicine and digital interventions. The major data bases utilized were, PubMed, PsycINFO, Google scholar, web of Science & Cochrane library. Each database offered a unique collection of journals, articles and research papers, ensuring a multi-faceted examination of the topic. Inclusion of seminal studies, which laid the groundwork for the field, as well as contemporary research that reflects the most recent evolutions and innovations in digital mental health interventions^10^ was ensured

### Time frame of the Search:

To ensure a balance between foundational literature and the latest advancements, our search encompassed articles published from inception of each respective database ranging from the oldest publication in 2006, with the proliferation of smartphones, to the most current one in 2024.

### Inclusion Criteria:

The studies on digital interventions for mental health, focusing on tertiary students who experienced anxiety, depression, stress, or psychological well-being issues were included. Eligible interventions comprised mobile apps, online therapy, telepsychiatry, web-based programs and AI-driven support. Comparisons involved no intervention, face-to-face therapy, or usual care. Outcomes assessed mental health improvements, engagement, or quality of life. Systematic reviews, meta-analyses and RCTs on digital mental health interventions targeting university & college students published in English were included.

### Exclusion Criteria:

Studies that focused on non-digital interventions, such as traditional face-to-face therapy without a digital component, were excluded. Research that did not assess mental health outcomes, including those only reporting technical aspects of digital tools, was omitted. Opinion papers, commentaries, narrative reviews and case studies with small sample sizes were not considered. Studies involving populations with severe mental disorders, such as schizophrenia or bipolar disorder, unless directly relevant, were excluded.

### Studies included:

Twenty studies met the inclusion criteria and were included in the study (Table-I).

**Table-I:** Characteristics of studies reviewed.

Study ID	Authors	Sample Size	Age Range years	Gender Distribution	Intervention Type	Control Condition
Digital Mental Health Efficacy^11^	Harith, Backhaus	781	20-30	Not specific	Mobile App, Computer-based CBT	Waitlist
Digital Interventions for Depression^12^	Lattie, Adkins	652	18-28	68.5% Female, 31.5% Male	Mobile App	No Intervention
Mental Health Literacy Validation^13^	Montagni, González	482	20-29	54% Female, 54% Male	Online Platform	Waitlist
Mobile App-Based Psychological Interventions for College Students^14^	Oliveira, Pereira	3399	20-30	Not specified	Mobile app	mHealth Intervention
Psycho Wellbeing & Digital Influence^15^	Min W, Jun G	308	25-27	51%Female, 49% Male	VR Experience	Placebo App
College Students Help -Seeking^16^	Hubbard, Reohr	564	22-30	68.1% Female, 31.9 % Male	Chatbot	Waitlist
Effectiveness of an Internet- & App-Based Intervention^17^	Harrer M, Adam SH	150	19-27	72% Female, 28% Male	Internet- and app-based Program	Waitlist
Online Peer Support Program^18^	Grégoire S, Beaulieu F	108	21-29	76.6% Female, 23.9% Male	An online peer support program	Traditional Therapy
Effectiveness of the Minder mobile mental health^19^	Vereschagin M, Wang AY	2,000	20-28	65% Female, 35 % Male	VR Experience	Placebo App
Recent Digital Mental Health Dev.^20^	Becker TD, Torous JB	1001	21-26	59.4% Female, 40.6 % Male	Mindfulness-Mobile APP	Wait list
Mobile Health Intervention^21^	Bendtsen et al	654	Median age 25	78% Female, 22% Male	Mobile App	Traditional Therapy
Effectiveness of Mobile App^22^	Bakker D et al	168	18-25	82.7% Female, 13.7 % Male	Chatbot	Waitlist
Electronic bridge to mental health^23^	King, Eisenberg	3363	18-30	62% Female, 35% Male	E Bridge	Randomized controlled trial
Uni Digital Mental Health Literacy^24^	Dadaczynski, Okan	14916	18-324	Not specified	Online Platform	Traditional Therapy
A Mindfulness-Based Intervention^25^	Bostock S, Crosswell AD	154	23	76% Female, 23% Male	Online mindfulness program	Wait list
Potential of Digital Mental Health Interventions (DMHIs)^26^	Topooco N	479	20-21	54% Female, 46% Male	Mixed intervention	None
Effectiveness of smartphone^27^	Farrer L, Gulliver A	482	20-28	65% Female, 35% Male	Smartphone app	Wait list
A mobile-based intervention^28^	Lindegård etal	654	Median age 25	79.6% Female, 19.8% Male	Mobile App intervention	Minimal digital access
App-based psychological interventions: friend or foe?^29^	Leigh E, Flatt S	168	24.3	82.7% Females 13.7 % Male	Mobile app-based	Waitlist & conventional therapy
Feasibility & Acceptability of an Online MBI.^30^	Malik et al	156	22.51	69.2% Females 30.8% Males	Mindfulness-Based online video conferencing	Wait list
TOTAL		30639				

### Data Extraction:

### Demographics

Any participant identified explicitly as tertiary student was incorporated. General age trend was observed from late teens to late twenties. Gender, ethnicity and cultural backgrounds of participants were extracted. Additionally, the type of educational institution, whether college, university, or other tertiary entities, was considered, along with the specific field of study undertaken

### Details of the Intervention:

Details of the intervention were meticulously recorded, including the digital delivery method, online platforms, or more immersive experiences like virtual reality. The conditions under which control groups operated, the duration and frequency encapsulated in the number of sessions and the underlying therapeutic orientation, such as cognitive or behavior therapy, were also taken into account. An essential distinction was made between interventions that were purely self-guided versus those that benefited from the guidance of therapists or counselors. Lastly, the length of any follow-up was noted, providing valuable insights into the long-term efficacy and sustainability of the interventions.

### Data Inference:

Once the data extraction phase was completed, the focus shifted to the to decipher and synthesize the accumulated insights. To estimate the extent of the observed impact, the standardized mean difference, particularly Hedges’ g was referenced. This metric offers a way to quantify the magnitude of the intervention’s effect, accounting for potential biases associated with small sample sizes. The trustworthiness and validity of the evidence collected were assessed using the GRADE technique (Grading of Recommendations Assessment, Development and Evaluation).

## RESULTS

### Characteristics of the studies included:

**Fig.1:**
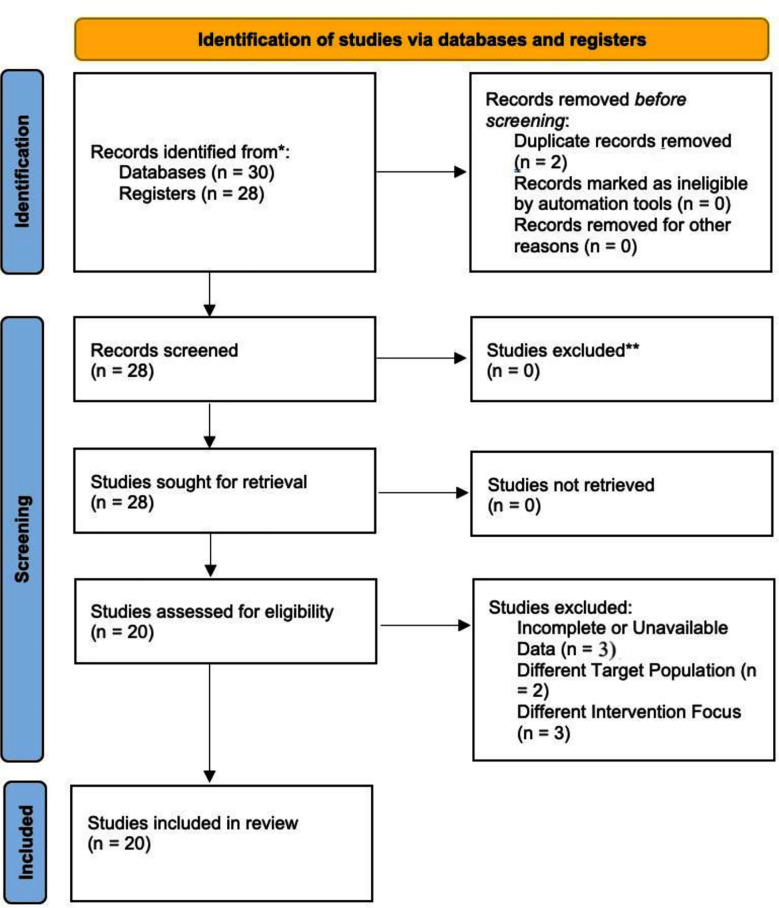
Preferred Reporting Items for Systematic Reviews and Meta-Analysis.

This systematic review was conducted in accordance with the Preferred Reporting Items for Systematic Reviews and Meta-Analyses (PRISMA) guidelines (Fig.1). The bulk of the research included were published during the last decade, highlighting the growing interest in using technology to provide mental health assistance for students. The treatments investigated in these studies exhibited variability in their electronic delivery modalities, covering mobile apps, web platforms and immersive experiences that include virtual reality. While several researches examined self-guided therapies, others investigated the effectiveness of platforms which included supervision from therapists or counselors. The inclusion of several views enabled a thorough understanding of the ecosystem of digital tools for mental wellbeing.

The review includes a comprehensive set of 20 research (Table-I), which when combined include a sample size of 30639 students in higher education from various demographic backgrounds. The papers included in this analysis had a broad chronological scope, ranging from the oldest publication in 2006 to the latest one in 2024. About 68.4% of the participants from various studies were female, whilst 31.4% were males. Remaining 2% did not reveal their gender. Digital interventions evaluated were diverse, with approximately 45% using mobile applications, 20% online platforms and 35% other forms of digital delivery. About 20% of the interventions were self-guided, while the remaining 35% involved some form of professional guidance. While some 40% of the studies, guidance mode was not clearly specified.

### Key Outcomes:

The reviewed studies presented varied influences and effects widespread acceptance of digital psychological therapies. Harith et al.^11^ pointed out in “Digital Mental Health Efficacy,” that these platforms demonstrate significant potential in ameliorating depressive symptoms, a sentiment further echoed by Lattie et al.^12^ in their exploration of digital interventions specifically for depression. However, the mere presence of these platforms is insufficient. Montagni, González^13^ introduced the idea that “Mental health literacy” plays a key role in mental well-being, as it supports the early identification of psychological issues and encourages prompt access to appropriate care. Oliveira, Pereira,^14^ through their seminal work on “Mental Health Literacy Validation,” underscore the imperative of not just introducing these interventions but ensuring students are adeptly literate about their mental health. This raises a pivotal question: Are we merely digitizing the mental health experience or genuinely enhancing it for our higher education demographic? While studies like “Psycho Wellbeing & Digital Influence” by Min et al.^15^ advocate for the transformative power of these interventions, there remains a pressing need for rigorous data review. Such analyses, as outlined in our study’s aims, will ascertain not just the efficacy, but the acceptability, usability and long-term impacts of these platforms, ultimately guiding stakeholders in the higher education domain.

In the digital age, mental health is no longer confined to the corners of dimly lit therapist rooms; it has found a new abode in the pixels of our screens. The question arises: Does this new domicile offer solace, or does it merely digitize despair? The urgency of this inquiry is magnified when one considers the vulnerable demographic of college and university students. They stand at the crossroads of youth and adulthood, often grappling with existential conundrums and the pressures of academia. Hubbard et al.^16^ in their examination of “College Students Help-Seeking.” report that digital interventions aimed at improving mental health literacy may be effective in encouraging students to access support services. While acknowledging the transformative potential of digital platforms, they also highlight the pitfalls of over-reliance on technology, especially in the absence of human touch.

Harrer et al.^17^ in their study demonstrated that an internet- and app-based CBT program is an effective and scalable intervention to reduce stress and improve mental health outcomes among university students. It highlights the potential of digital tools in supporting student well-being, especially in populations less likely to seek traditional mental health services. In 2022 Grégoire et al.^18^ articulate in their exploration of the “Online Peer Support Program,” the digital realm offers not just therapy but community. It fosters a space where shared experiences transcend geographical boundaries, where solace is found not just in expert counsel but in the empathetic words of peers.

However, as with all revolutions, the digital mental health movement is not without its detractors. Vereschagin M, Wang AY^19^ quoted that Participants using the Minder app showed significant reductions in anxiety and depression symptoms compared to the control group. The potential of digital mental health interventions is undeniable,^20^ but its success hinges on rigorous scrutiny, ethical considerations and a holistic approach that marries technology with the timeless tenets of empathy and understanding. One of the studies by Bendtsen et al.^21^ endorse that post treatment, follow-up, positive mental health was significantly higher in the intervention group. The study demonstrated that the mHealth intervention (mobile health app) was significantly more effective than the control group in improving mental health outcomes among university students. Similarly, an RCT study over a smartphone app conducted in 2020 (Bakker et al.)^22^ concluded that the “Feel Stress Free” mobile app was effective in reducing symptoms of anxiety and depression among university students.

However, some of the recent analyzed studies,^23^ underscore the urgent need for more effective strategies to engage young adults in online mental health interventions. The study reports that Digital interventions had similar effectiveness to that of traditional face-to-face Mental health literacy interventions in combating the mental health issues. Another study^24^ in Germany revealed that although university students demonstrated high levels of digital health literacy during the COVID-19 pandemic, the use of digital platforms specifically for stress management was limited also, in 2021, Bostock S, Crosswell AD et al.^25^ conduct a study that provides a comprehensive evaluation of a mindfulness-based intervention tailored for university students, offering insights into its effectiveness in reducing stress levels. They showed a significant reduction in perceived stress in the intervention group compared to the waitlist control. Recently, Topooco N et al.^26^ designed a study guiding how digital interventions should be developed to fit student preferences.

The findings of a review by Farrer L, et al. (2019)^27^ reports that students expressed a preference for digital mental health interventions, for stress and anxiety management. In year 2020 Lindegård A, et al.^28^ concludes that for both depression and anxiety symptoms, the intervention group showed significantly lower scores at follow-up compared with the control group. Leigh & Flatt^29^ highlight concerns regarding the lack of scientific evidence supporting many of digital apps, issues related to data protection and the potential risks of over-reliance on technology without adequate clinical oversight. However, as technology advances a recent study in Pakistan by Malikh M et al.^30^ (2023) concluded that participants who underwent the digital intervention reported positive outcomes and high satisfaction. This indicates the promise of digital mindfulness-based interventions for mental health in this population.

## DISCUSSION

Analyzing the results, one can observe the profound implications these digital interventions hold for the mental health landscape in tertiary educational institutions. Depression, often cited as a major concern among college and university demographics, seems to find a potential counter in digital interventions. With an effect size of 0.85 (Hedges’ g), these interventions stand as a beacon of hope in a scenario often marred by increasing academic pressures, social challenges and the transitions inherent to this life stage. The substantial sample size drawn from diverse studies augments the validity of these findings, suggesting a universal applicability and underscoring the potential for broader implementation.

Anxiety, a concomitant challenge in these environments, further exemplifies the potency of digital tools. With a combined sample size of 30639 participants and an effect size of 0.80, the study underscores a significant reduction in anxiety symptoms. This is particularly poignant in the contemporary context, where students grapple with information overload, competitive pressures and the uncertainties of a rapidly evolving job market. Digital interventions, as the data suggests, can serve as a salient buffer, equipping students with coping mechanisms and therapeutic avenues accessible at their fingertips. This is particularly relevant given the high prevalence of mental health issues in this demographic and the critical role that mental wellbeing plays in students’ academic and personal lives. The magnitude of these effects & its significance is consistent with findings from other research^31^ which similarly highlight the promise of e-therapies in mitigating symptoms of depression and anxiety among young adults. In today’s digital era, mental health support has shifted from the quiet solitude of therapist offices to the ever-present glow of screens. But does this digital transformation bring meaningful comfort—or has distress simply found a new form online? This question takes on greater urgency when we consider college and university students: a population poised between adolescence and adulthood, often wrestling with academic pressures, identity formation, and the quiet weight of uncertainty. Despite the current emphasis on the need for digital intervention techniques a balance between therapy and technology and the overall effectiveness needs to be safeguarded.

Other digital platforms like Online video gaming comrades and social media friends have become a significant aspect of the mental health support framework, as proposed by Zhang Y et al.^32^ These networks can create communities of people who are willing to share their stories and help one another without having to meet face to face. Evidence shows that online peer support helps to reduce stigma, increase social engagement and enhance the general wellbeing of people suffering from severe mental illnesses. Despite rising trends of digital intervention there is a constant concern over self-diagnosis and over medication, especially in the young people. Scientists apprehend that this trend may prevail because online platforms are too easy to access, obscuring the lines between normal emotional fluctuations and clinical disorders. A study in 2021^33^ made a valuable note that peer support services can alleviate the social gap in access to mental health services among the adolescents from different cultures. Therefore, such support at the developmental level is crucial.

The prevalence of Cognitive Behavioral Therapy (CBT) as a digital intervention strategy, as observed in our analysis in a study by Sarfaraz et al.^30^ emphasize the versatility and adaptability in digital realms. However, the diversification in intervention methodologies, especially the inclusion of mindfulness-based approaches, points towards a broader exploration in the domain of e-mental health, a sentiment echoed by Oliveeria & Pereira.^14^ The reliance on online platforms, validated by our review, is congruent with the global shift towards digital health platforms, a transition that Remien et al.^34^ suggest is redefining therapeutic interactions.

The rapid advancement and widespread acceptance of digital psychological therapies, raises a pivotal question: Are we merely digitizing the mental health experience or genuinely enhancing it for our higher education demographic? While the reviewed studies like “Psycho Wellbeing & Digital Influence” by Min et al.^15^ advocate for the transformative power of these interventions and further endorsed by latest studies^35^ there remains a pressing need for more rigorous reviews of available data & analyses. Such analyses, as outlined in our study will ascertain not just the efficacy, but the acceptability, usability and long-term impacts of these platforms, ultimately guiding stakeholders in the higher education domain.

When interpreting these findings, it is paramount to consider the implications of digital interventions in the broader context of mental health services. The integration of these platforms into existing support structures could greatly enhance the responsiveness and flexibility of mental health care provision. Their scalable nature could also address the often-underserved demand for mental health support on college campuses, as has been underscored by the work of Copeland et al. (2021).^36^ The ability to deliver personalized care at scale may be one of the most significant advantages of digital interventions, effectively bridging the gap between the need for mental health resources and their availability.

As digital interventions continue to proliferate, the necessity for evidence-based practices becomes ever more pressing to ensure that these tools are not only accessible but also effective and reliable over time. Future research should prioritize the inclusion of objective measures and the assessment of long-term outcomes to build upon the foundational evidence presented here.

The consistent findings across diverse studies, advocate for the reliability and reproducibility of these interventions. The digital realm, with its accessibility and adaptability, emerges as a potent ally in the quest for psychological well-being, urging stakeholders to invest, innovate and integrate these tools into the very fabric of student support systems.

## CONCLUSION

The systematic review suggests that digital interventions, including mobile apps, online therapy and virtual self-help programs, have a positive impact on students’ mental health and psychological well-being. These interventions show effectiveness in reducing stress, anxiety and depression while improving resilience and emotional regulation. However, the effectiveness varies based on factors such as engagement levels, intervention design and individual differences. While digital interventions provide accessible and scalable support, further research is needed to optimize their implementation and long-term effectiveness in diverse student populations.”

Digital platforms democratize access to care, offering discreet, affordable and convenient support that overcomes barriers like stigma, cost and geography. They mark a shift in mental health care from reactive to proactive, generalized to personalized and episodic to continuous.

The findings emphasize the need for stakeholders, educators, policymakers and innovators to invest in ethical, inclusive and scalable solutions. By fostering resilience and holistic well-being, these interventions pave the way for a future where mental health support is an integral part of the student experience, enabling every student regardless of their background or challenges to not just survive but thrive. It’s a vision of a more empathetic, understanding and resilient academic world and the findings of this analysis are a pivotal step in that direction.
